# Brain Tumors in Pregnancy: A Review of Pathophysiology, Clinical Management, and Ethical Dilemmas

**DOI:** 10.3390/cancers17233854

**Published:** 2025-11-30

**Authors:** Muratbek A. Tleubergenov, Daniyar K. Zhamoldin, Dauren S. Baymukhanov, Assel S. Omarova, Nurzhan A. Ryskeldiyev, Aidos Doskaliyev, Talshyn M. Ukybassova, Serik Akshulakov

**Affiliations:** 1 Department of Brain Neurosurgery, JSC “National Centre for Neurosurgery”, Astana 010000, Kazakhstan; 2 Department of Gynecology, JSC “University Medical Center”, Astana 010000, Kazakhstan; 3 JSC “National Centre for Neurosurgery”, Astana 010000, Kazakhstan

**Keywords:** brain tumor, pregnancy, neurosurgery, fetal outcome, maternal survival

## Abstract

Background: Central nervous system (CNS) tumors during pregnancy are rare but present significant diagnostic, therapeutic, and ethical challenges. These include both primary and metastatic lesions, which share overlapping clinical features and management complexities. Their clinical course is influenced by gestational physiological changes, which can mask symptoms and delay diagnosis, thereby increasing maternal and fetal risks. Objective: This review aims to synthesize current evidence on the epidemiology, pathophysiology, clinical presentation, diagnostic strategies, treatment options, prognosis, and ethical considerations related to CNS tumors in pregnant patients. Methods: A comprehensive literature review was conducted, including retrospective and prospective studies, clinical guidelines, and systematic reviews focusing on brain and spinal tumors diagnosed during pregnancy. Particular attention was given to the impact of gestational age, tumor histology, and maternal condition on treatment outcomes. Results: Hormone-sensitive tumors such as meningiomas and prolactinomas may exhibit accelerated growth during pregnancy due to elevated progesterone and prolactin levels. Diagnosis is often delayed due to symptom overlap with normal gestational changes. MRI without contrast remains the imaging modality of choice. Glucocorticoids and selected chemotherapy agents can be cautiously used depending on gestational age. Surgical resection, particularly in the second trimester, has been shown to be safe and effective in appropriate clinical scenarios. Multidisciplinary coordination is essential. Prognosis varies based on tumor type and timing of intervention, with maternal survival prioritized in high-risk situations. Ethical management hinges on patient autonomy, informed consent, and proportionality of medical interventions. Conclusions: CNS tumors during pregnancy require early recognition, individualized treatment planning, and ethical vigilance. Multidisciplinary collaboration is vital to optimizing outcomes for both mother and fetus. Future efforts should focus on developing standardized protocols and expanding evidence through multicenter studies.

## Simple Summary

Intracranial tumors diagnosed during pregnancy represent a rare but clinically significant condition that poses risks to both maternal and fetal health. Clinical manifestations such as headache, nausea, and visual disturbances frequently overlap with physiological changes in pregnancy, often resulting in delayed diagnosis. Certain neoplasms, including meningiomas and prolactinomas, may demonstrate accelerated growth under the influence of hormonal and hemodynamic alterations. These challenges are observed in both primary and metastatic brain tumors, which often present with similar symptoms and management dilemmas during pregnancy. This review consolidates current evidence regarding the pathophysiological mechanisms, diagnostic approaches, and therapeutic strategies for managing brain tumors in pregnant patients. A comprehensive understanding of these aspects facilitates timely recognition, optimization of imaging and treatment protocols, and multidisciplinary decision-making aimed at improving maternal and fetal outcomes.

## 1. Introduction

Central nervous system (CNS) tumors in pregnant women represent a rare but clinically significant condition, associated with substantial risks to both the mother and the fetus. Although these neoplasms account for less than 2% of all oncological diagnoses, they may present during the reproductive years, requiring a multidisciplinary approach for accurate diagnosis and effective management [1]. The estimated incidence of brain tumors during pregnancy ranges from 3 to 6 cases per 100,000 deliveries, with gliomas, meningiomas, and pituitary adenomas being the most frequently encountered histological types [2,3].

Physiological changes during pregnancy play a crucial role in modulating the clinical course of CNS tumors. Increased circulating blood volume, fluid retention, and elevated levels of estrogen and progesterone are particularly relevant in hormone-sensitive tumors such as meningiomas and prolactinomas. Cowppli-Bony et al. reported that the incidence of meningiomas in women of reproductive age is nearly twice as high as in men, likely due to the expression of estrogen and progesterone receptors in tumor tissues [4].

Diagnosis is often delayed due to the non-specificity of symptoms. Headache, vomiting, and seizures are frequently misattributed to normal pregnancy-related changes, leading to a diagnostic delay that can exceed three weeks on average [5]. Nonetheless, timely identification and an individualized treatment strategy can significantly improve maternal and perinatal outcomes.

Therapeutic approaches range from close monitoring and pharmacologic management to surgical resection, with chemotherapy and radiotherapy considered in select cases. The choice of intervention depends on gestational age and the teratogenic risks associated with various treatment modalities [6]. Anesthetic management also plays a critical role; according to Abd-Elsayed et al., decisions regarding general versus regional anesthesia, as well as the feasibility of simultaneous craniotomy and cesarean delivery, must be guided by comprehensive neurosurgical and obstetric assessment [3].

In summary, CNS tumors during pregnancy constitute a multifaceted clinical challenge involving complex diagnostic, therapeutic, and ethical considerations. This literature review aims to synthesize current knowledge on the epidemiology, pathogenesis, diagnosis, management, and prognosis of brain tumors in pregnant patients.

## 2. Methodology

This study was conducted as a narrative review, designed to synthesize and interpret existing knowledge regarding the pathophysiology, clinical management, and ethical dimensions of central nervous system tumors in pregnancy. The narrative review approach was selected because it allows for integration of heterogeneous data, theoretical perspectives, and clinical experiences that cannot be captured through systematic or quantitative methods.

An iterative literature search was performed between January and October 2025 using the electronic databases PubMed, Scopus, Web of Science, and Google Scholar. The following combinations of search terms and Boolean operators were used: “brain tumor” AND “pregnancy”; “central nervous system neoplasm” OR “CNS tumor” AND “gestation”; “meningioma”, “glioma”, “pituitary adenoma” AND “pregnancy management”; “neurosurgery” AND “pregnancy” AND (“maternal outcomes” OR “fetal outcomes”).

Manual reference tracking (“snowballing”) of key reviews and relevant clinical reports was performed to identify additional sources not captured by database indexing. The search strategy was iterative, with adjustments made as new evidence and themes emerged during data collection.

To ensure comprehensiveness, no restrictions were initially applied to study design, but only peer-reviewed articles in English were included in the final synthesis.

Inclusion and Exclusion Criteria

Included sources comprised:

- Peer-reviewed journal articles, reviews, and clinical guidelines published in English from 1990–2025;
- Both retrospective and prospective studies, as well as systematic reviews addressing CNS tumors diagnosed during pregnancy;
- Publications describing diagnostic, therapeutic, prognostic, or ethical aspects of neuro-oncological management.

Excluded were:

- Non-peer-reviewed materials;
- Animal studies or in vitro experiments without direct clinical relevance;
- Studies focusing exclusively on non-pregnant populations.

Data Selection and Analysis

Each included paper was analyzed for:

- Study design and patient population;
- Tumor type and clinical presentation;
- Diagnostic and therapeutic approach;
- Maternal and fetal outcomes;
- And ethical or decision-making aspects.

A qualitative thematic synthesis was applied, organizing findings into the following core domains:

- Epidemiology of brain tumors in pregnancy;
- Pathophysiological mechanisms;
- Clinical presentation and diagnostic features;
- Therapeutic management (conservative and surgical);
- Maternal and fetal prognosis;
- Ethical dilemmas in clinical practice.

Recurring concepts, divergences, and knowledge gaps were identified and summarized narratively.

Reflexivity and Rigor

Given the interpretive nature of narrative reviews, the authors acknowledge potential subjectivity in data interpretation. To enhance rigor:

- The literature selection was independently verified by multiple co-authors with backgrounds in neurosurgery and obstetrics;
- The analytical process followed transparent documentation of search terms, databases, and inclusion decisions;
- Findings were cross-validated with systematic reviews and meta-analyses when available.

This reflexive and multidisciplinary approach ensured balanced and contextually grounded synthesis of evidence.

Limitations of the Method

The narrative format does not aim for exhaustive coverage of all available literature. Selection bias and variability in study design across sources may limit reproducibility. However, this flexible approach allows for deeper exploration of clinical, physiological, and ethical nuances that structured meta-analyses may overlook. Future research should complement narrative synthesis with systematic data aggregation and quantitative meta-analytic validation.

## 3. Epidemiology of Brain Tumors in Pregnancy

Brain tumors during pregnancy are a rare yet clinically significant condition requiring a multidisciplinary diagnostic and therapeutic approach. The incidence of malignant CNS neoplasms among women of reproductive age (20–39 years) is estimated at 2.0 to 3.2 per 100,000 per year, with a corresponding mortality rate ranging from 0.5 to 1.1 per 100,000 [7]. In pregnant populations specifically, a retrospective analysis by Isla et al. identified 7 cases of brain tumors among 126,413 pregnancies, yielding an incidence of approximately 5.5 per 100,000 [8].

A systematic review by Rodrigues et al. compiled 454 cases of CNS tumors diagnosed during pregnancy. The most common tumor types included gliomas (~30%), meningiomas (~27%), pituitary adenomas (8–10%), ependymomas (5–10%), schwannomas (~5%), medulloblastomas (~3%), as well as metastatic and vascular tumors (each accounting for 3–5%) [9].

These findings are consistent with data from national registries such as SEER and CBTRUS. The overall incidence of CNS tumors in women is 24.77 per 100,000, compared to 20.34 per 100,000 in men. Among women aged 15 to 39 years, the incidence is 10.94 per 100,000. Primary brain tumors in this population are predominantly meningiomas (37.6%) and gliomas (27%) [10].

Meningiomas are the prototypical hormone-sensitive brain tumors. Among women of childbearing age, the incidence nearly doubles that of men (6.5 vs. 3.05 per 100,000), attributed to the expression of PRs and ERs [11]. The presence of hormonal receptors in both meningiomas and astrocytomas has been validated by multiple studies [8,12,13]. Roelvink et al. reported accelerated tumor growth during the third trimester, potentially driven by enhanced vascularization and the hormonal milieu of late pregnancy [14].

Pituitary adenomas, particularly prolactinomas, comprise 8–10% of all CNS tumors observed in pregnancy [9]. According to Molitch, clinically significant tumor enlargement occurs in only 1.4% of women with microadenomas and typically does not require surgical intervention. In contrast, untreated macroadenomas present with symptomatic growth in 26.2% of cases, often necessitating surgery or resumption of bromocriptine therapy. In women who received treatment prior to conception, the risk of symptomatic progression is reduced to just 3% [15].

Despite their low absolute frequency, brain tumors in pregnancy warrant heightened clinical attention due to their high impact on maternal and fetal health. The histological distribution is characterized by a predominance of hormone-sensitive tumors—gliomas, meningiomas, and prolactinomas—which tend to become clinically manifest during the second and third trimesters [16,17]. Given the nonspecific nature of presenting symptoms, a high index of clinical suspicion is essential among obstetricians, neurologists, and general physicians [5,17].

Epidemiological data on the incidence of brain tumors during pregnancy are highly fragmented and vary significantly depending on the source, methodology, and database used. The lack of standardized reporting criteria, the mixing of population-based (per 100,000 women per year) and event-based (per 100,000 pregnancies) measures, as well as the diagnostic limitations specific to pregnancy, make these estimates inherently uncertain and complicate direct comparisons. To interpret the prevalence of CNS tumors in pregnant women accurately, it is essential to account for differences in measurement units: general incidence is typically expressed per 100,000 women per year, whereas tumor detection rates during pregnancy are reported per 100,000 pregnancies. These figures cannot be directly compared without additional adjustment, as they reflect different aspects of the same population. Given the heterogeneity and methodological inconsistency across available studies, we opted not to present a summary table, as it may inaccurately imply comparability where none exists.

## 4. Pathophysiology of Brain Tumors During Pregnancy

The growth of CNS tumors during pregnancy is driven by a complex interplay of hormonal, hemodynamic, and vascular factors. These mechanisms are particularly relevant in previously asymptomatic lesions that become clinically evident under the physiological changes in gestation.

Meningiomas, which account for approximately 36% of all primary brain tumors, exhibit pronounced hormonal sensitivity and occur more frequently in women of reproductive age [12,18]. The study by Isla et al. demonstrated that tumor progression in pregnant patients is often clinically significant during the third trimester—the period of peak hormonal activity [8].

Hemodynamic adaptations of pregnancy also contribute to tumor progression. A 30–50% increase in cardiac output leads to enhanced cerebral perfusion and hypervascularization, particularly affecting tumors with rich vascular networks such as meningiomas [7,19]. These tumors may increase in volume due to edema and hyperemia without evidence of malignant transformation, as confirmed in clinical observations in approximately 30% of patients [20].

**Figure 1:**
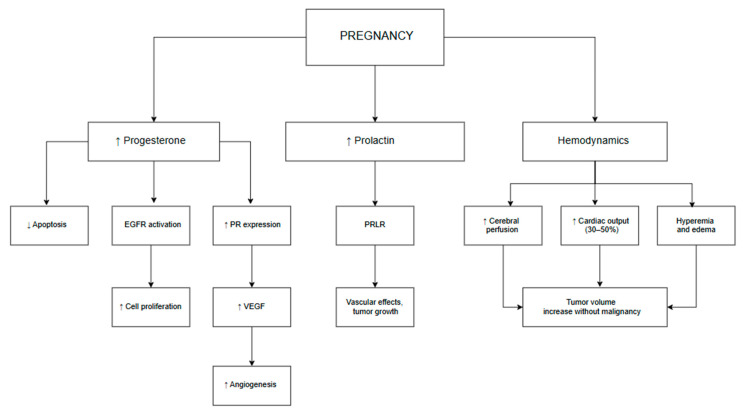
Pregnancy-associated endocrine and vascular mechanisms in meningioma growth. The schematic illustrates hormonal (progesterone, prolactin) and hemodynamic (increased cardiac output, hyperemia) factors contributing to tumor volume expansion, particularly in the third trimester, without evidence of malignant transformation.

Hormonal regulation plays a central role in the pathogenesis of meningiomas. PRs are expressed in 70–95% of tumors, while ERs are identified in only 20–30% of cases [21]. In pregnant women, PR expression in meningioma tissue is particularly elevated, suggesting a direct stimulatory effect of progesterone [8]. During the third trimester, progesterone levels rise more than tenfold, promoting angiogenesis via VEGF, activating EGFR, and inhibiting apoptosis [22]. Taken together, the combined effects of elevated progesterone levels, receptor-mediated angiogenesis, and pregnancy-related hemodynamic adaptations create a permissive environment for tumor progression. An overview of these mechanisms is depicted in Figure 1, emphasizing their clinical relevance, particularly during the third trimester.

**Figure 2:**
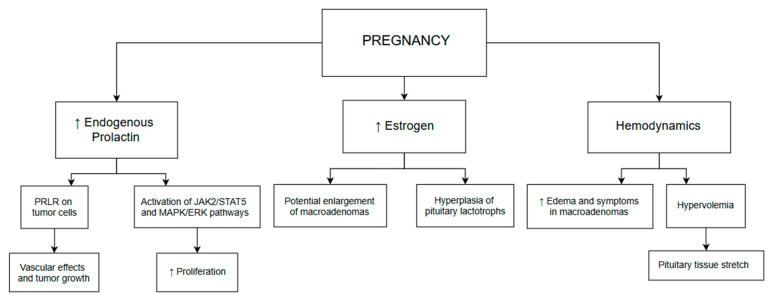
Pregnancy-associated endocrine and vascular mechanisms in prolactinoma growth. The schematic demonstrates the role of endogenous prolactin and estrogen in activating PRLR-mediated signaling (JAK/STAT, MAPK/ERK), enhancing proliferation, and promoting lactotroph hyperplasia. Hemodynamic changes, including hypervolemia and pituitary tissue stretch, further contribute to the clinical manifestation of macroadenomas, particularly in the third trimester and postpartum.

Prolactin is another potential mediator of tumor growth. In a review by Laviv et al., 61% of meningiomas in pregnant patients were diagnosed during the third trimester or immediately postpartum, implicating a possible role of prolactin in tumor dynamics [23]. Its effects are likely mediated by vascular mechanisms and the presence of prolactin-specific receptors within tumor tissue. Collectively, these endocrine and hemodynamic alterations are illustrated in Figure 2, emphasizing the clinical significance of prolactin-driven tumor growth during late pregnancy and postpartum.

Gliomas and astrocytomas may also increase in size during pregnancy, though hemodynamic changes—such as hypoxemia, hypercapnia, and hypervolemia—appear to play a more dominant role in their progression [9]. Nevertheless, evidence suggests a possible involvement of prolactin in the pathophysiology of these tumors as well. Prolactin has been shown to activate JAK2/STAT5 and MAPK/ERK signaling cascades Via the PRLR, potentially contributing to the growth of glioblastomas [24].

In summary, the progression of brain tumors during pregnancy is mediated by multiple mechanisms, including hormonal influences (progesterone, prolactin), vascular changes, and alterations in systemic hemodynamics. Understanding these pathophysiological features is essential for timely diagnosis, monitoring, and management of affected patients.

## 5. Clinical Presentation of Brain Tumors During Pregnancy

The clinical manifestations of brain tumors in pregnant women are highly variable and are frequently masked by the physiological changes in gestation. This overlap contributes to delayed recognition and diagnosis, with an average lag of 3 to 6 weeks from symptom onset to definitive diagnosis [17]. According to Yust-Katz et al., symptoms most often emerge during the second and third trimesters, when hormonal and hemodynamic alterations peak and can precipitate decompensation of intracranial dynamics [16].

Headache is the most commonly reported symptom, present in 78–85% of patients with CNS tumors during pregnancy [17,18]. Unlike gestational migraine, tumor-related cephalalgia tends to be focal, worsens at night or in the early morning, and is frequently accompanied by nausea, vomiting, altered consciousness, and poor response to standard analgesics. Bonfield and Engh emphasize that persistent, progressive headache during the second or third trimester should prompt urgent neuroimaging [7].

Seizures occur in 30–55% of pregnant patients with brain tumors, particularly in cases involving gliomas and metastatic lesions [16,17]. In approximately 20% of cases, seizures debut before 20 weeks’ gestation [8]. High-grade gliomas are associated with the greatest risk, often presenting with acute neurologic deterioration and necessitating urgent delivery [17].

Visual disturbances—including decreased visual acuity, diplopia, and hemianopia—are reported in 25–30% of patients. These symptoms are especially common in tumors affecting the optic chiasm and hypothalamic region, such as meningiomas, pituitary adenomas, and craniopharyngiomas [9,15]. In some cases, visual impairment may be the sole initial manifestation.

Focal neurological deficits depend on tumor localization. Lesions of the cerebellum or brainstem can cause ataxia, dysarthria, and weakness; frontal lobe tumors may present with aphasia or seizures; parasagittal meningiomas are often associated with sensory disturbances and facial asymmetry. According to Roelvink et al., 62% of pregnant women with meningiomas developed focal deficits that worsened during pregnancy [13].

Signs of increased ICP, such as intractable vomiting and papilledema, are seen in 15–25% of cases, particularly in patients with posterior fossa tumors or obstructive hydrocephalus [17,20].

Diagnostic errors are common. In up to 40% of cases, initial symptoms are misinterpreted as preeclampsia, toxicosis, or migraine [20]. As reported by Terry et al., the most frequent indications for hospital admission were seizures and altered consciousness; however, neuroimaging was often delayed or omitted [25].

A systematic analysis by Rodrigues et al. showed that symptom onset peaked in the second (38%) and third trimesters (47%), with only 15% of cases presenting in the first trimester [9]. These trends reflect the increasing impact of hormonal and vascular changes as pregnancy progresses.

## 6. Diagnostic Considerations in Brain Tumors During Pregnancy

Diagnosing brain tumors during pregnancy presents a complex and clinically significant challenge. Physiological changes associated with gestation can obscure or mimic tumor-related symptoms, while limitations in imaging modalities further complicate timely recognition. The central diagnostic issue is differentiating between tumor-related and pregnancy-related manifestations, as intracranial neoplasms often present with symptoms that overlap with normal gestational physiology.

According to De Haan et al., in 40% of cases, headache was the sole presenting symptom in the absence of focal neurological deficits, leading to diagnostic delays and clinical misinterpretation [26]. Similarly, Lynch et al. reported that symptoms in approximately 40% of patients were misattributed to migraine or eclampsia, with definitive diagnosis typically made around the 28th week of gestation [20].

MRI without contrast is the gold standard for evaluating CNS tumors in pregnant patients. It is considered safe at any stage of pregnancy, free from ionizing radiation, and offers high sensitivity for space-occupying lesions [17,22,27]. According to the UCSF Radiology “CT and MR Pregnancy Guidelines,” exposure to MRI during the first trimester has not been associated with an increased risk of harm to the fetus or during early childhood, further supporting its safety profile in this population [28]. In clinical practice, MRI without contrast was used in 83% of cases involving pregnant women with brain tumors, as reported by Nguyen et al. [29].

The use of GBCA is limited to strict indications. A large cohort study by Ray et al., involving 1.42 million pregnancies, found that gadolinium exposure was associated with higher rates of stillbirth (3.7% vs. 0.7%) and neonatal skin disorders (7.5% vs. 2.4%) [21,27]. Current guidelines from the American College of Radiology recommend gadolinium only when non-contrast imaging is insufficient for diagnostic or therapeutic decision-making [21]. Its use is more permissible during the second and third trimesters, particularly when assessing tumor edema, vascular invasion, or mass effect [19].

Cranial CT is considered a secondary diagnostic option, reserved for emergency situations such as suspected intracranial hemorrhage or significant cerebral edema. The radiation dose from a head CT is less than 0.01 Gy—well below the teratogenic threshold of 5–10 Gy [28,30]. In the study by Nguyen et al., CT was utilized in 12% of pregnant patients with brain tumors, primarily under urgent clinical conditions. Nonetheless, its use during the first trimester should be avoided unless absolutely indicated [7,29].

Special attention must be given to clinical red flags that warrant immediate neuroimaging. These include persistent and progressive headaches (up to 65%), seizures (40–60%), visual disturbances (up to 30%), vomiting beyond the first trimester (25–35%), and episodes of altered consciousness (10–15%) [7,31].

In conclusion, the early and accurate diagnosis of intracranial tumors during pregnancy requires a high level of clinical vigilance, adherence to modern diagnostic protocols, and close collaboration among obstetricians, neurologists, neurosurgeons, and radiologists.

## 7. Conservative Management of Brain Tumors During Pregnancy

Pharmacologic management of CNS tumors in pregnant patients requires an individualized approach, taking into account gestational age, tumor histology and grade, and overall maternal condition. The main pharmacologic strategies include anti-edema therapy, anticonvulsant treatment, and, in select cases, chemotherapy.

Glucocorticoids are a cornerstone of supportive medical therapy. According to Laviv et al., most pregnant patients with intracranial tumors present with significant cerebral edema requiring immediate steroid administration prior to neurosurgical intervention [23]. In addition to their anti-edema effects, corticosteroids such as dexamethasone promote fetal lung maturation, making them especially valuable in obstetric care when preterm delivery is anticipated [23].

Corticosteroids are effective in alleviating symptoms such as headache, nausea, and visual disturbances by reducing vasogenic edema, though they do not exhibit direct antitumor activity. Arias et al. note that in clinically stable patients, glucocorticoids can substantially improve quality of life [32].

Common antenatal steroid regimens include two 12 mg intramuscular injections of betamethasone administered 24 h apart, or four 6 mg doses of dexamethasone given every 12 h. However, as Kemp et al. highlight, these protocols lack robust clinical validation and are based primarily on observational data rather than randomized trials [33].

Steroid use should be cautious, particularly in light of emerging evidence on long-term risks, including fetal growth restriction and neurodevelopmental delays [34]. In cases of progressive maternal deterioration, surgical intervention should be prioritized over prolonged steroid therapy.

Chemotherapy is contraindicated during the first trimester due to a high risk of congenital anomalies (up to 25%) and miscarriage (up to 30%) [26,29]. After organogenesis is complete, chemotherapy may be considered in the second trimester (teratogenic risk < 5%) and third trimester (risk < 2%), though the risk of intrauterine growth restriction increases with gestational age. In patients with aggressive tumors and poor prognosis, chemotherapy may serve as a temporizing measure until fetal viability is achieved [26].

Drug selection must be approached with caution. Temozolomide, an FDA category D agent, is both teratogenic and mutagenic and is contraindicated during pregnancy. Cisplatin (also category D) has been used in the second and third trimesters with reassuring data from retrospective studies. Vincristine (category D) may be considered after 14 weeks of gestation. In contrast, bevacizumab (category C) is contraindicated due to its anti-angiogenic effects, which may disrupt placental development and embryogenesis [29].

In summary, pharmacologic management must strike a careful balance between maternal benefit and fetal risk. Medication use should be guided by a multidisciplinary team, including a neuro-oncologist, obstetrician, clinical pharmacologist, and, when necessary, a neonatologist. Conservative therapy is preferable in clinically stable patients when radical treatment can be safely deferred until fetal viability is achieved [26,29].

## 8. Surgical Management of Brain Tumors During Pregnancy

Surgical intervention remains the primary treatment modality for CNS tumors in pregnant patients, particularly in cases of cerebral compression, elevated intracranial pressure, seizure syndromes, altered consciousness, or signs of brain herniation [7,35]. The therapeutic approach is guided by the urgency of the clinical condition, gestational age, and the presumed histological nature of the tumor.

Laviv et al. reported that among 148 pregnant patients with meningiomas, 132 (89%) required surgical treatment [23]. The majority of surgeries were performed in the second trimester (74 cases, 50%), with no maternal mortality and a perinatal mortality rate of 2.7%. Surgeries in the first trimester were less frequent (18 cases, 12%) and similarly showed no maternal deaths, although the perinatal loss rate reached 11.1%. In the third trimester, 40 procedures (27%) were carried out without adverse fetal outcomes. The mean gestational age at the time of neurosurgery was 23 weeks, and craniotomy with gross total resection was the most common approach [23].

Cohen-Gadol et al. further support the safety of neurosurgical intervention during pregnancy [36]. Among 34 pregnant women with intracranial tumors, 19 (56%) underwent surgery during gestation. Of these, 74% received craniotomies, 10% underwent stereotactic biopsies, and 16% required shunt placement. There were no instances of maternal death, severe neurological complications, or fetal morbidity [36].

Combined cesarean section and neurosurgical intervention in the third trimester has also been documented. Laviv and colleagues described three such cases, in which simultaneous cesarean delivery and craniotomy were performed, resulting in survival of all neonates and timely maternal treatment [35]. Similar conclusions have been drawn by other authors, who advocate this combined strategy as a balanced approach that optimally safeguards both maternal and fetal outcomes in late pregnancy [29].

Emergency surgical intervention is indicated in cases of rapid neurological deterioration, including declining consciousness, focal deficits, or status epilepticus. In a study by Tewari et al., 8 of 10 women with malignant brain tumors required preterm delivery between 27 and 32 weeks; 4 of these underwent immediate craniotomy [37]. This approach was associated with favorable fetal outcomes even in aggressive clinical scenarios [37]. Beyond individual series and reviews, a comparative synthesis of reported cases highlights the heterogeneity of surgical decision-making in pregnant women with brain tumors. The compiled literature demonstrates variability in timing of intervention, delivery mode, tumor histology, and both maternal and perinatal outcomes. Table 1 provides an overview of reported surgical strategies and their clinical consequences, underscoring the complexity of balancing oncological safety with maternal and fetal prognosis.

**Figure 3:**
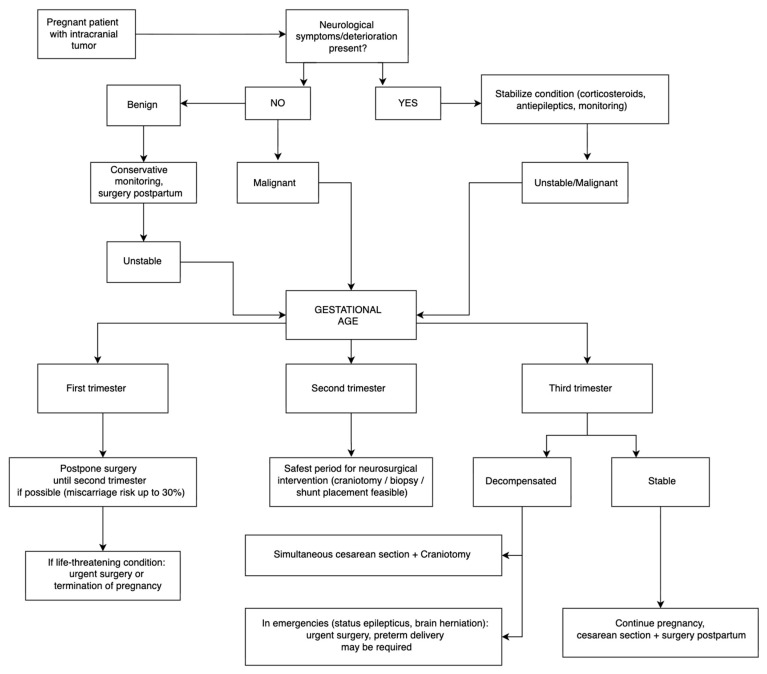
Clinical decision-making algorithm for pregnant patients with intracranial tumors. The scheme incorporates neurological deterioration, tumor type, maternal stability, and gestational age to guide management, including stabilization measures, timing of surgery (preferably in the second trimester), and indications for combined cesarean delivery and neurosurgical intervention in late pregnancy.

A more structured approach to treatment planning in pregnant patients with intracranial tumors has been proposed by Zohdy et al., yet its practical relevance lies not in the stepwise branching shown in Figure 3, but in the underlying clinical principles it highlights [5]. The most decisive parameter is maternal neurological status: progressive deficits, uncontrolled seizures, rising intracranial pressure, or signs of herniation mandate urgent stabilization and often expedited neurosurgical intervention, regardless of gestational age [5]. Tumor biology also plays a central role. Benign, slow-growing lesions in stable patients can typically be managed conservatively until postpartum, whereas malignant or clinically unstable tumors require earlier intervention, with the therapeutic window shifting according to trimester.

Importantly, the algorithm underscores nuances not apparent from the visual scheme alone. In the first trimester, the priority is avoiding fetal loss (miscarriage risk up to 30% [35]), so surgery is reserved for life-threatening scenarios. The second trimester provides the safest balance between maternal anesthesia tolerance and fetal stability, making it the optimal period for craniotomy, biopsy, or shunt placement. In the late second and third trimester, maternal deterioration necessitates coordinated management with obstetrics; depending on fetal maturity, simultaneous cesarean section and neurosurgical intervention may allow timely maternal treatment without compromising neonatal outcomes [29,35]. Thus, Figure 3 is not intended as a rigid protocol but as a synthesis of these clinical priorities—neurological urgency, tumor aggressiveness, gestational timing, and maternal stability—which together shape individualized, trimester-specific decision-making.

Several operative techniques hold special value. Awake craniotomy may be employed for tumors in eloquent brain regions. A review by Mofatteh et al. described nine such cases in pregnant patients, primarily with gliomas, all resulting in full maternal and fetal survival [39]. Another strategy is preoperative embolization of hypervascular meningiomas. A meta-analysis by Chen et al. showed that embolization reduced intraoperative blood loss by an average of 65 mL and shortened operative time by 38 min without increasing complications [40].

In summary, surgical treatment of brain tumors during pregnancy is feasible and can be safe when guided by careful preoperative planning, appropriate timing, and multidisciplinary coordination. The most favorable outcomes are consistently observed when surgery is performed during the second trimester, with no maternal mortality and minimal fetal risk [23,36,41]. Typical management strategies, optimal timing, and associated maternal and fetal outcomes for the most common tumor types are summarized in Table 2.

## 9. Maternal and Fetal Prognosis in Brain Tumors During Pregnancy

The diagnosis of CNS tumors during pregnancy is associated with significant risks to both maternal and fetal outcomes. Prognosis depends on multiple factors, including tumor histology, gestational age at diagnosis, presence of complications such as intracranial hypertension, seizures, or hemorrhage, and the necessity for urgent intervention.

In a large retrospective study by Terry et al., involving 860 pregnant women with CNS tumors among more than 19.7 million hospitalizations in the United States, malignant brain tumors were associated with a 143-fold increase in maternal mortality risk [25]. The study also revealed a more than threefold increase in preterm birth rates, a nearly threefold increase in intrauterine growth restriction, and a 6.4-fold increase in cesarean delivery compared to controls [25]. Even in cases of benign tumors, obstetric complications were more common: preterm delivery occurred 2.3 times more frequently, hyperemesis gravidarum 2.8 times more frequently, and cesarean section rates nearly tripled. In spinal cord tumors, cesarean delivery was 3.9 times more common. Notably, neurosurgical procedures were performed in 33% of cases; however, no significant increase in maternal or perinatal mortality was observed, supporting the safety of surgical intervention when guided by appropriate risk stratification [25].

Prognostic evaluation incorporates several clinical scoring systems. Maternal functional status is assessed using the KPSS, postoperative recovery via the GOS, and neonatal condition through the Apgar score. In cases of tumor control and term delivery, Apgar scores at 1 and 5 min are generally ≥7, indicating satisfactory neonatal adaptation. Key factors negatively influencing fetal prognosis include malignancy of the tumor, need for emergency delivery, and fetal hypoxia secondary to seizures or intracranial decompensation. Maternal outcome is largely dependent on tumor stage, the extent of therapeutic intervention, and gestational age. In patients with marked symptoms or indications for neurosurgery, cesarean section is typically the preferred mode of delivery.

Additional insights on hemorrhagic complications are provided by Leffert et al., who reported an incidence of subarachnoid and intracerebral hemorrhage of approximately 6 per 100,000 deliveries [42]. Despite the traditionally high lethality rates associated with these events (up to 10% for subarachnoid and 20% for intracerebral hemorrhage), pregnant patients demonstrated more favorable outcomes. The risk of death was five times lower in cases of subarachnoid hemorrhage and nearly two times lower for intracerebral hemorrhage compared to the general population. Furthermore, the likelihood of ambulatory function at discharge was higher among pregnant patients, suggesting improved neurological recovery when timely treatment is provided [42].

In conclusion, with comprehensive, multidisciplinary management, pregnancy complicated by CNS tumors can result in favorable maternal and fetal outcomes—even under high-risk conditions.

## 10. Ethical Considerations in the Management of Brain Tumors During Pregnancy

The management of CNS tumors in pregnant women raises profound ethical challenges that go beyond the standard principles of biomedical ethics. Decisions often involve urgent life-threatening conditions, uncertain prognoses, and conflicting duties to both the mother and fetus. The key ethical tension lies in balancing maternal autonomy and beneficence with fetal protection while navigating diverse cultural, legal, and institutional frameworks.

A woman’s right to make decisions about her own body remains the ethical cornerstone of modern obstetric practice. The ACOG explicitly affirms that a competent pregnant woman retains full decision-making authority, even when her choices may endanger fetal life [43]. By contrast, the FIGO emphasizes the need to weigh fetal interests once viability is reached, promoting a “dual-patient” model where both mother and fetus warrant moral consideration [44]. The ESMO adopts an intermediate stance, supporting aggressive maternal treatment even during pregnancy if maternal prognosis is otherwise compromised, provided that risks are transparently communicated [45]. Meanwhile, in the UK context, case-based guidance (e.g., from the Nuffield Council on Bioethics) suggests that when maternal and fetal interests diverge and consensus cannot be reached, referral to a hospital clinical ethics committee may be appropriate [46].

Real-world neuro-oncological cases illustrate the complexity of these principles. For example, when a malignant glioma is diagnosed in the first trimester and urgent chemoradiation is indicated, proceeding with treatment implies near-certain fetal loss. In such cases, ACOG and ESMO permit maternal therapy after informed consent, prioritizing maternal survival, whereas some national frameworks may restrict such interventions before 12 weeks of gestation. Conversely, when a slow-growing meningioma causes visual decline in the third trimester, conservative management until fetal maturity aligns with FIGO’s emphasis on fetal beneficence. These contrasting paradigms underscore the importance of contextual ethics—applying universal principles within legal and cultural realities.

The issue of pregnancy termination in CNS malignancy remains ethically contentious. While ACOG discourages termination purely for theoretical maternal benefit, ESMO and FIGO consider it ethically permissible in cases of poor maternal prognosis, severe neurological deterioration, or when teratogenic therapy cannot be postponed. Quantitative data support this approach: Dotters-Katz et al. found that pregnancy termination rarely improves maternal outcomes except in high-grade gliomas diagnosed before 20 weeks’ gestation [47]. Therefore, most international guidelines advocate maternal-first prioritization—stabilization of the mother to maximize the chance of survival for both.

Special challenges arise when the patient’s decision-making capacity is compromised due to cognitive or neurological impairment (e.g., aphasia, psychosis, or altered consciousness). In such cases, decisions must rely on legal representatives, previously expressed preferences, or multidisciplinary ethics consultation. The principle of justice demands equitable access to care, regardless of gestational age or prognosis, ensuring that pregnant women are not denied neurosurgical or oncological interventions available to non-pregnant patients [48].

Ultimately, ethical management of CNS tumors in pregnancy requires more than abstract adherence to moral principles. It demands dynamic, case-sensitive reasoning that integrates international guidelines, respects cultural context, and prioritizes open communication between clinicians and patients. Only through such reflexive, multidisciplinary deliberation can care align with both moral integrity and medical best practice.

## 11. Limitations

This review is subject to several important limitations. First, the available literature on brain tumors during pregnancy is limited by small sample sizes, heterogeneous methodologies, and a predominance of retrospective case reports and case series. These study designs are prone to selection bias and may not adequately capture the full clinical spectrum or long-term outcomes. Second, epidemiological data vary widely across studies due to inconsistent reporting standards and differences in diagnostic criteria, limiting the comparability of incidence and prevalence estimates. Third, much of the therapeutic evidence—particularly regarding pharmacologic safety, timing of surgical intervention, and fetal outcomes—is derived from observational data rather than randomized controlled trials, thereby limiting the strength of clinical recommendations. Fourth, ethical considerations were discussed in a theoretical context and may not reflect the complexity of real-world clinical decision-making in diverse cultural and legal settings. Finally, this review does not include a formal meta-analysis or statistical synthesis, as the heterogeneity of reported cases precluded quantitative aggregation. Future multicenter prospective studies and standardized registries are needed to address these gaps and improve evidence-based care for pregnant patients with CNS tumors.

## 12. Conclusions

Central nervous system tumors diagnosed during pregnancy represent not only a complex medical condition but also an ethical challenge, necessitating a careful, multidisciplinary approach. Although rare, their potential threat to maternal and fetal life demands early detection and prompt clinical decision-making.

Current evidence indicates that pregnancy may accelerate the growth of certain tumor types—particularly hormone-sensitive neoplasms such as meningiomas and prolactinomas. At the same time, the physiological changes in gestation can obscure typical symptoms, complicating timely diagnosis. This underscores the need for heightened clinical vigilance, especially in cases of atypical headache, seizures, or visual disturbances.

Treatment strategies must be strictly individualized, considering gestational age, tumor histology, biological aggressiveness, and the maternal clinical condition. In most scenarios, a collaborative effort involving neurosurgeons, obstetricians, anesthesiologists, oncologists, and clinical ethicists is key to formulating optimal management plans that minimize risks to both mother and fetus.

Looking ahead, there remains a pressing need to develop standardized clinical protocols and conduct multicenter studies to refine prognostic assessments, evaluate therapeutic effectiveness, and establish evidence-based guidelines for the management of CNS tumors during pregnancy.

## Figures and Tables

**Table 1:** Comparative table of reported surgical management of brain tumors during pregnancy and maternal-perinatal outcomes.

Author	Number of Patients	Trimester of Operation	Intervention	Delivery	Histology	Maternal Outcome	Perinatal Outcome
[38]	1	I	VP shunt for hydrocephalus after TAB	TAB at 9 weeks GA	Melanoma MTS	Mother succumbed to malignant cerebral edema.	N/A
	3	II	Resection of cerebellar mass; palliative RT + chemo postpartum	C/S at 30 weeks GA	Breast cancer MTS	Mother and child are alive at the time of study termination.	
			Chemo at 9 weeks GA; craniotomy at 16 and 27 weeks GA; chemo at 22 weeks GA; RT at 30 weeks GA	C/S at 32 weeks GA.	Breast cancer MTS	Mother and child are alive and healthy at 6 weeks follow-up.	
			Craniotomy + GTR of frontal met at 24 weeks GA; postop RT (GKRS) at 25 weeks GA	C/S at 36 weeks GA.	Breast cancer MTS	Mother and child are alive and well at 3.5 years follow-up.	
	1	III	GTR at 24 weeks GA (2nd preg) + postop RT (5 fx) with maternal–fetal shielding	N/A	Lung cancer MTS	N/A	N/A
	3	Postpartum	Chemo during preg; post fossa decompression + RT + SRS; lapatinib + capecitabine postpartum	Forceps delivery at 37 weeks GA	Breast cancer MTS	Mother and child are alive at the time of study termination.	
			Resection of temporal mts after delivery of 1st preg	C/S at 36 weeks GA	Recurrent breast cancer MTS	N/A	The first child is alive and well at 5 years of age.
			Emergency craniotomy for raised ICP + cerebellar lesion resection postpartum	C/S at 38 weeks GA	Alveolar soft tissue sarcoma MTS	Mother and child are alive and well at 10 months follow-up.	
[39]	2	I	AC + GTR	C/S at 34 weeks GA	Giant cell glioblastoma (WHO grade IV)	N/A	Fetus stable post-op; delivered at 34 weeks; healthy at 5 mo FU
			AC + TR	N/A	Meningioma	Symptom resolution, stable hemodynamics	Fetus stable post-op
	5	II	AC + TR	N/A	Glioma	Patient deceased 16 months after craniotomy	Viable infant with normal Apgar score
			AC + TR	Vaginal at term	Anaplastic oligoastrocytoma (WHO grade III)	No deficits	Fetus stable post-op
			AC + TR	Vaginal at term	Anaplastic astrocytoma (WHO grade III)	N/A	Fetus stable post-op
			AC + TR	N/A	N/A	N/A	Fetus stable post-op
			AC + TR	N/A	Astrocytoma (Grade II/III)	N/A	Fetus stable post-op
	1	III	AC + TR—Two general anesthesia tumor debulking during the same pregnancy at 16 weeks and 28 weeks gestation	Vaginal (twins) at term	Anaplastic astrocytoma (WHO grade III)	No complication	Twins delivered post-op 4th day under spinal anesthesia.
[36]	3	I	Stereo Bx TAB + XRT and chemo	TAB	Grade III astro	TAB at 4 weeks in preparation for XRT and chemo	5 by GOS
			VP shunt → TAB + XRT	TAB (2 weeks after shunt)	Intraventricular tumor (no tissue)	Therapeutic abortion at 2 weeks after shunt placement	5 by GOS
			Craniotomy + Resection + XRT	N/A	GBM	N/A	5 by GOS
	6	II	Craniotomy + Resection + XRT	N/A	GBM	N/A	4 by GOS
			Stereo Bx + XRT; chemo postpartum	NSVD	Grade III astro	Normal	5 by GOS
			Craniotomy + Resection	NSVD	Grade II astro	Normal	5 by GOS
			Craniotomy + Resection	Pregnancy in progress	Meningioma	N/A	N/A
			Ventriculoatrial shunt → TAB + XRT/chemo	TAB 6 days after shunt placement	Infiltrative pineal tumor (no tissue)	TAB	4 by GOS
			Emergency C/S → Craniotomy + Resection 12 h later	Emergency C/S	Meningioma	Normal	5 by GOS
	1	III	XRT	NSVD	Thalamic tumor (GBM at autopsy)	Normal	1 by GOS

AC—awake craniotomy; Bx—biopsy; C/S—cesarean section; FU—follow-up; GA—gestational age; GKRS—Gamma Knife radiosurgery; GOS—Glasgow Outcome Scale; GTR—gross total resection; NSVD—normal spontaneous vaginal delivery; RT (XRT)—radiotherapy; SRS—stereotactic radiosurgery; TAB—therapeutic abortion; TR—tumor resection; Chemo—chemotherapy; MTS—metastasis; astro—astrocytoma; GBM—glioblastoma multiforme.

**Table 2:** Summary of preferred management strategies and maternal–fetal outcomes in brain tumors during pregnancy.

Tumor Type	Preferred Management	Optimal Timing (Trimester)	Maternal Outcome	Fetal Outcome	Sources
Meningioma	Surgical resection if neurological deterioration; conservative otherwise	2nd trimester	Excellent (no maternal mortality in reviewed series)	Good (>95% live births)	[23]
Glioma	Case-by-case; surgery for high-grade or symptomatic lesions	2nd trimester	Variable (depends on grade)	Good if gestational age > 28 weeks	[37]
Pituitary adenoma	Medical management; surgery rare	3rd trimester or postpartum	Excellent	Excellent	[15]
Metastatic tumors	Palliative or combined management; chemo after 2nd trimester	Any (if indicated)	Favorable (depends on primary site)	Excellent	[38]
Overall	Multidisciplinary individualized approach	2nd trimester safest for surgery	Maternal survival ~95%	Fetal survival > 90%	Summary from current review

## Data Availability

No new data were created or analyzed in this study. Data sharing is not applicable to this article.

## Abbreviations

SEER	Surveillance, Epidemiology, and End Results
CBTRUS	Central Brain Tumor Registry of the United States
PR	Progesterone Receptor
ER	Estrogen Receptor
VEGF	Vascular Endothelial Growth Factor
JAK2/STAT5	Janus Kinase 2/Signal Transducer and Activator of Transcription 5
MAPK/ERK	Mitogen-Activated Protein Kinase/Extracellular Signal-Regulated Kinase
PRLR	Prolactin Receptor
ICP	Intracranial Pressure
GBCA	Gadolinium-Based Contrast Agents
KPSS	Karnofsky Performance Status Scale
GOS	Glasgow Outcome Scale
ACOG	American College of Obstetricians and Gynecologists
FIGO	International Federation of Gynecology and Obstetrics
ESMO	European Society for Medical Oncology
